# Apical but not sub-apical hyphal compartments are self-sustaining in growth

**DOI:** 10.1007/s10482-020-01383-9

**Published:** 2020-01-09

**Authors:** Martin Tegelaar, George P. A. van der Lans, Han A. B. Wösten

**Affiliations:** Microbiology, Department of Biology, Utrecht University, Padualaan 8, 3584 CH Utrecht, The Netherlands

**Keywords:** Fungus, Hyphae, Compartment, Apical growth, Branching

## Abstract

It was recently demonstrated that apical compartments of *Aspergillus niger* hyphae are self-sustaining in growth. This was shown by assessing the growth rate of individual hyphae before and after dissection of the second compartment. Using the same methodology, it is here demonstrated that single apical compartments of the septate fungi *Penicillium chrysogenum* and *Schizophyllum commune* as well as the 500-µm-apical region of the non-septate fungus *Rhizopus stolonifer* are also self-sustaining in growth. In contrast, single 2nd compartments (obtained by dissection of the first and third compartment) of the septate fungi or the region between 500 and 1000 µm from tips of *R. stolonifer* were severely impacted in their growth rate. In addition, it is shown that existing or newly formed branches originating from the 2nd compartments function as a backup system for hyphal growth when the apical part of the hypha of the three studied fungi is damaged. Together, it is concluded that the apical compartments/zones of the studied fungi are self-sustaining in growth. In contrast, the subapical region is not self-sustaining but functions as a backup once the apical zone is damaged. This back up system is relevant in nature because the apices of hyphae are the first to be exposed to (a)biotic stress conditions when entering an unexplored substrate.

## Introduction

Filamentous fungi form mycelia that consist of a network of hyphae. These hyphae extend at their apices and branch sub-apically. Hyphae of mucoromycetes are generally non-compartmentalized, while those of ascomycetes and basidiomycetes are divided by septa. These septa have a 50–500 nm wide central pore flanked by invaginations of the cell wall lined with plasma membrane (Shatkin and Tatum 1959; Moore and McAlear 1962; Lew 2005; Steinberg et al. 2017). By plugging the pores, intercompartmental and interhyphal cytoplasmic mixing is interrupted (Bleichrodt et al. 2012, 2015a). Intact growing hyphae of *Aspergillus oryzae* (Bleichrodt et al. 2012), *Aspergillus niger* (Tegelaar et al. 2019), and *Zymoseptoria tritici* (2017b) plug their pores with Woronin bodies, while hyphae of the basidiomycete *Schizophyllum commune* close their pores with the septal pore cap (van Peer et al. 2010). Environmental conditions impact incidence of septal closure in *S. commune* (van Peer et al. 2009)*, A. oryzae* and *A. niger* (Tegelaar et al. 2019). Low glucose levels reduce plugging incidence in *S. commune*, while presence of antibiotics, heat shock, and hypertonic shock promote septal closure (van Peer et al. 2009). Plugging incidence of septa of *A. oryzae* is affected by heat treatment, low pH conditions, C-starvation and N-starvation, while that of *A. niger* is affected by exposure to heat, high pH, and hypertonic conditions (Tegelaar et al. 2019). Incidence of septal closure in *Aspergillus* also increases in time. Half of the newly formed apical septa are open, while septa flanking the 10th compartment are always closed (Bleichrodt et al. 2012, 2015a). From this and the fact that septal closure abolishes bulk mixing of cytoplasm it was concluded that hyphal compartments transform from a unicellular to a multicellular system (Bleichrodt et al. 2015a). Yet, selective transport is still possible via transporters in the septal plasma membrane (Bleichrodt et al. 2015b).

The fact that apical compartments of *Aspergillus* hyphae form a unicellular system is in line with the concept of the peripheral growth zone. This zone defines the number of compartments that are needed for a hypha to maintain its growth rate (Trinci 1971). The peripheral growth zones of *A. niger* and *Penicillium chrysogenum* would consist of 11 and 13 intact compartments and 8660 µm for the non-septate hyphae of *Rhizopus stolonifer.* Notably, it was recently shown that apical compartments of *A. niger* are self-sustaining (Tegelaar and Wösten 2017), thus reducing its peripheral growth zone to one compartment. This difference was explained by the fact that Trinci (1971) determined the growth rate of colonies before and after damaging the mycelium at different lengths from the outer part of the colony, while Tegelaar and Wösten (2017) assessed growth after dissection of individual hyphae. Using the latter methodology, it is here shown that single apical compartments of the ascomycete *P. chrysogenum* and the basidiomycete *S. commune* and the first 500 µm of hyphae of *R. stolonifer* are also self-sustaining in growth. In contrast, single 2nd compartments (obtained by dissection of the first and third compartment) of septate fungi or the region between 500 and 1000 µm from the tip of *R. stolonifer* were severely impacted in their growth rate.

## Materials and methods

### Strains and growth conditions

Strains N402 of *A. niger* (Bos et al. 1988), *P. chrysogenum* Wisconsin 54-1255 (Stauffer 1961), *R. stolonifer* CBS 112376 and *S. commune* H4-8 (matA43 matB41; FGSC 9210 (Ohm et al. 2010) were used in this study. The former two fungi were grown at 30 °C in water-saturated air in the dark, while the latter two fungi were grown at 25 °C and 50% humidity in~ 750 lx white light (C65 100 mA 5730, NS12 spectrum, Valoya, Helsinki, Finland). *R. stolonifer* plates were sealed with cellophane. Spores of *A. niger* and *P. chrysogenum* were harvested in 10 ml 0.9% NaCl (w/v), 0.05% (v/v) Tween-20 from 7-day-old cultures grown in 9 cm Petri dishes on complete medium (CM). This medium consisted of minimal medium (MM; 0.6% NaNO_3_, 0.15% KH_2_PO_4_, 0.05% KCl, 0.0 5% MgSO_4_ 7H_2_O, and 0.2 ml l^−1^ Vishniac solution (Vishniac and Santer 1957), pH 6.0) supplemented with 0.5% yeast extract, 0.2% casamino acids, 25 mM maltose and 1.5% agarose. Spores of *R. stolonifer* were obtained as described above, but harvested after 3 days. *S. commune* was grown on polycarbonate membranes (Whatman Cyclopore™, 76 mm diameter 0.1 µm pore size, Osmonics; GE Water Technologies) placed on *S. commune* minimal medium (SCMM; Dons et al. 1979) solidified with 1.5% agarose. After 3 days the *S. commune* mycelium was macerated in 50 ml SCMM for 1 min at high speed with a Waring Blender. The homogenated mycelium was transferred to a 50 ml Greiner tube and was incubated for 24 h at 25 °C in the dark. Spores and micro-colonies obtained in these ways were used to inoculate glass bottom dishes (Cellview™ cell culture dishes, PS, 35/10 mm, Greiner Bio-One, Frickenhausen, Germany). To this end, the dishes and medium were pre-warmed to 60  °C. 0.5 µL spore solution (50,000 spores), or one *S. commune* micro-colony, was placed on a glass coverslip (18 mm in diameter and 0.16–0.19 mm thick) and left to dry. Spores were allowed to dry completely, while micro-colonies were allowed to dry until no more medium was visible around the micro-colony. 30 µL pre-warmed MMA (MM supplemented with 25 mM maltose and 1% agarose) or SCMMA (SCMM with 1% agarose), was added to the middle of the glass bottom dish, and immediately brought in contact with coverslip-adhered *A. niger, P. chrysogenum* and *R. stolonifer* spores or *S. commune* micro-colonies, respectively. After the layer of medium had solidified, 2 ml of corresponding liquid medium (i.e. (SC)MM supplemented with 25 mM maltose) was pipetted on top of the coverslip. Laser dissection was performed after 48 h of growth.

### Microscopy

Hyphae were dissected using a PALM Microbeam system (laser power 35%, focus 57%) linked to an Observer.Z1 inverted microscope (Carl Zeiss AG, Oberkochen, Germany) and a CCD color camera (AxioCam ICc 1, Carl Zeiss AG, Oberkochen, Germany). Growth rate of hyphae and branches was recorded every 5 min during a 15 min period prior to dissection. In addition, it was assessed whether septal pores, if present, were open or closed at the moment of dissection. Septa were classified as open when cytosol was leaking through the septal pore upon dissection (Bleichrodt et al. 2012). Hyphal growth after dissection was recorded every 5 min for 45 min for *P. chrysogenum* and *S. commune* hyphae cut in the 2nd compartment. Growth from hyphae cut in the apex or in both the apex and 3rd compartment in *A. niger* and *P. chrysogenum* was recorded every 30 min during a 4 h period after dissection, with non-dissected hyphae serving as control. For *R. stolonifer*, growth was recorded every hour during a 4 h period after dissection, with non-dissected hyphae serving as control. Single 2nd compartments of *S. commune* were followed for > 7 h using the PALM RoboSoftware time-lapse function. Width and length of compartment pre- and post-dissection were also recorded, as well as translocation (µm) of vacuoles after cutting. From these parameters the post-dissection volume of the apical compartment was calculated (Tegelaar and Wösten 2017).

### Statistics

Experiments were performed using at least biological triplicates (i.e. independent cultures grown at different days), each with at least 7 technical replicates (i.e. repeated measures within a biological replicate). Single 2nd compartments of *S. commune* that were followed using time-lapse had 11 biological replicates and no technical replicates. *T* tests or Mann–Whitney U tests were carried out to determine differences in growth rate between treatment and control apical compartments. Differences in hyphal growth or volume before and after cutting were determined using paired sample t-tests, followed by correlation and regression analyses. Analysis of bimodality was performed as described (Vinck et al. 2011).

## Results

### Closure of a septum, or the presence of a septum at all, is not a prerequisite for continued growth after sub-apical hyphal damage

Growth rate of hyphae of *P. chrysogenum, S. commune* and *R. stolonifer* was 79 ± 4, 173 ± 16 and 102 ± 20 µm h^−1^, respectively (for growth conditions see “Materials and methods” section) (Table 1). Mean hyphal growth rate was reduced by 8, 4, and 71% after dissection within the second compartment of *P. chrysogenum* and *S. commune* or 500 µm from the apex in the case of *R. stolonifer* (called pseudo-apical compartment from now on), respectively (Table 1). This mean hyphal growth rate was calculated including the 9, 4, and 40% of the (pseudo-)apical compartments of *P. chrysogenum, S. commune* and *R. stolonifer* that stopped growing after dissection, respectively. Growth rate of single apical compartments of *P. chrysogenum* and *S. commune* was not affected when only hyphae were taken into account that remained growing after cutting. Notably, even apical hyphal fragments of the coenocytic *R. stolonifer* that continued growing did not show a significant reduction in extension (Table 1). The apical septum of all dissected *S. commune* hyphae was open before cutting, while 21% of the *P. chrysogenum* apical septa were closed. More specifically, 29 and 17% of the apical septa were closed of the *P. chrysogenum* apical compartments that stopped and continued growing after dissection, respectively. Together, these findings show that closure of a septum, or the presence of a septum at all, is not a prerequisite for continued growth after dissection. Yet, the presence of a septum contributes to the survival of hyphae after injury.

**Table 1:** Length, width, and growth rate of the first compartment of *P. chrysogenum* and *S. commune* and the apical zone of *R. stolonifer* before and after laser dissection

	Length (µm)	Width (µm)	Growth rate before dissection (µm h^−1^)		Growth rate after dissection (µm h^−1^)	
			All hyphae	Growing hyphae after dissection	All hyphae	Growing hyphae after dissection
*P. chrysogenum* N = 353	243 ± 17 N = 144	4.5 ± 0.05 N = 174	79 ± 4 N = 353	79 ± 4 N = 320	73 ± 4 N = 353	80 ± 4 N = 320
*S. commune* N = 86	295 ± 17 N = 86	4.1 ± 0.16 N = 44	173 ± 16 N = 85	174 ± 15 N = 45	168 ± 20 N = 47	175 ± 20 N = 45
*R. stolonifer* N = 47	500 N = 34	3.6 ± 0.4 N = 24	102 ± 20 N = 14	74 ± 16 N = 7	30 ± 10 N = 34	65 ± 18 N = 18

Mean growth rate (± 95% confidence interval) before and after dissection is indicated of all dissected hyphae and of those hyphae that continued growing after laser dissection

On average, the (pseudo-)apical compartments that halted their growth after dissection lost 11, 51 and 69% of their cytoplasm in the case of *P. chrysogenum, S. commune* and *R. stolonifer,* respectively. In contrast, single apical fragments that continued their growth lost 1, 13 and 27% of their cellular volume, respectively. These data show that growth of the apical fragments of the septate fungi *P. chrysogenum* and *S. commune* are more sensitive to loss of volume when compared to *R. stolonifer.*

Subapical compartments assume peripheral growth after dissection of the apex.

Hyphae of *A. niger, P. chrysogenum,* and *S. commune* were dissected in the middle of the apical compartment, while *R. stolonifer* was cut at the very tip. Growth from the 2nd compartment, or, in the case of *R. stolonifer,* sub-apical to the damaged apex was recorded after laser dissection. These compartments of *A. niger, P. chrysogenum, S. commune* and the 500-µm-peripheral zone of *R. stolonifer* had formed lateral branches before dissection in 10, 18, 50 and 100% of the cases, respectively. The mean extension rate of these branches was 14.8 ± 3.7, 14.9 ± 3.2 and 148.2 ± 20.6 µm h^−1^ for *A. niger, P. chrysogenum* and *S. commune*, respectively (Table 2). No growth was observed from previously formed branches in *R. stolonifer*. After laser dissection of the apex of the main hypha, previously formed lateral branches originating from the subapical zone continued their growth in 71, 78, 100 and 2% for *A. niger, P. chrysogenum, S. commune* and *R. stolonifer* and had a mean extension rate of 9.5 ± 3.8, 13.9 ± 1.6, 132 ± 10.2 and 43.6 ± 23.8 µm h^−1^, respectively (Table 2). Thus, laser dissection of the apical zone of the studied fungi did not affect growth rate of branches that were previously formed from the 2nd compartment.

**Table 2:** Growth rate of existing and newly formed branches and the time period between dissection and the formation of new branches

Second compartment	Growth rate existing branches (µm h^−1^)		Growth rate newly formed branches (µm h^−1^)			Time between dissection and formation of new branches (min)		
	Intact hypha	Dissected hypha	Apical branch	Basal branch	Lateral branch	Apical branch	Basal branch	Lateral branch
*Aspergillus niger*								
Connected N = 109	14.8 ± 3.7 N = 7	9.5 ± 3.8 N = 5	21.6 ± 2.3 N = 30	N/A	16.1 ± 2.1 N = 21	77 ± 20 N = 30	N/A	59 ± 19 N = 21
Single N = 77	N.D.	14.1 ± 12.4 N = 1	9.1 ± 2.1 N = 31	6.6 ± 1.5 N = 25	10.9 ± 5.7 N = 7	69 ± 44 N = 31	165 ± 113 N = 25	69 ± 81 N = 7
*Penicillium chrysogenum*								
Connected N = 110	19.4 ± 4.4 N = 7	26.9 ± 6.0 N = 9	16.2 ± 1.3 N = 33	N/A	31.2 ± 5.4 N = 5	65 ± 10 N = 33	N/A	94 ± 30 N = 5
Single N = 56	N.D.	27.3 ± 8.9 N = 4	6.4 ± 1.9 N = 17	5.7 ± 1.6 N = 16	14.2 ± 9.9 N = 1	60 ± 31 N = 17	81 ± 19 N = 16	100 ± 172 N = 1
*Schizophyllum commune*								
Connected N = 59	148.2 ± 20.6 N = 4	132 ± 10.2 N = 25	56.3 ± 54.9 N = 7	N/A	100 ± 14.6 N = 26	180 ± 0 N = 7	N/A	74 ± 25 N = 26
Single N = 11	N.D.	11.4 ± 23.8 N = 4	17.8 ± 27.6 N = 5	9.9 ± 17.6 N = 4	6.5 ± 3.9 N = 6	566 ± 311 N = 5	346 ± 378 N = 4	508 ± 179 N = 6
*Rhizopus stolonifer*								
Connected N = 91	0	43.6 ± 23.8 N = 1	N/A	N/A	43.2 ± 6.8 N = 39	N/A	N/A	20 ± 18 N = 39
Single N = 47	N.D.	0	N/A	N/A	15.5 ± 14.4 N = 7	N/A	N/A	321 ± 436 N = 7

Hyphae of *A. niger, P. chrysogenum,* and *S. commune* were dissected in the middle of the apical compartment, while *R. stolonifer* was cut at the very tip (connected second compartments). Hyphae were also cut in the third (pseudo-)3rd compartment in the case of single (pseudo-)2nd compartments. Growth rates of lateral branches from single 2nd compartments before laser dissection was not determined (N.D.) because they were assumed to be similar to those of connected 2nd compartments. Apical and basal branches were not formed by *R. stolonifer* single pseudo-2nd compartments and basal branches were not formed for connected (pseudo-)2nd compartments (N/A)

After dissection of the apical compartment, newly formed branches appeared through the apical septum in 47, 57 and 10% of the hyphae of *A. niger, P. chrysogenum* and *S. commune*, respectively. These newly formed branches had a mean extension rate of 21.6 ± 2.3, 16.2 ± 1.3 and 56.3 ± 54.9 µm h^−1^ (Table 2). In contrast, newly formed lateral branches originating from the 2nd compartment had a mean extension rate of 16.1 ± 2.1, 31.2 ± 5.4 and 100 ± 14.6 µm h^−1^. After apical injury, *R. stolonifer* hyphae placed a septum at a mean distance of 69 ± 13 µm from the apical wound whenever growth was resumed by branches that were newly formed under this new septum. These branches had a mean growth rate of 43.2 ± 6.8 µm h^−1^ (Table 2). Together, data show that branches that had been formed before and after dissection have a similar growth rate after cutting the hyphae.

**Fig. 1:**
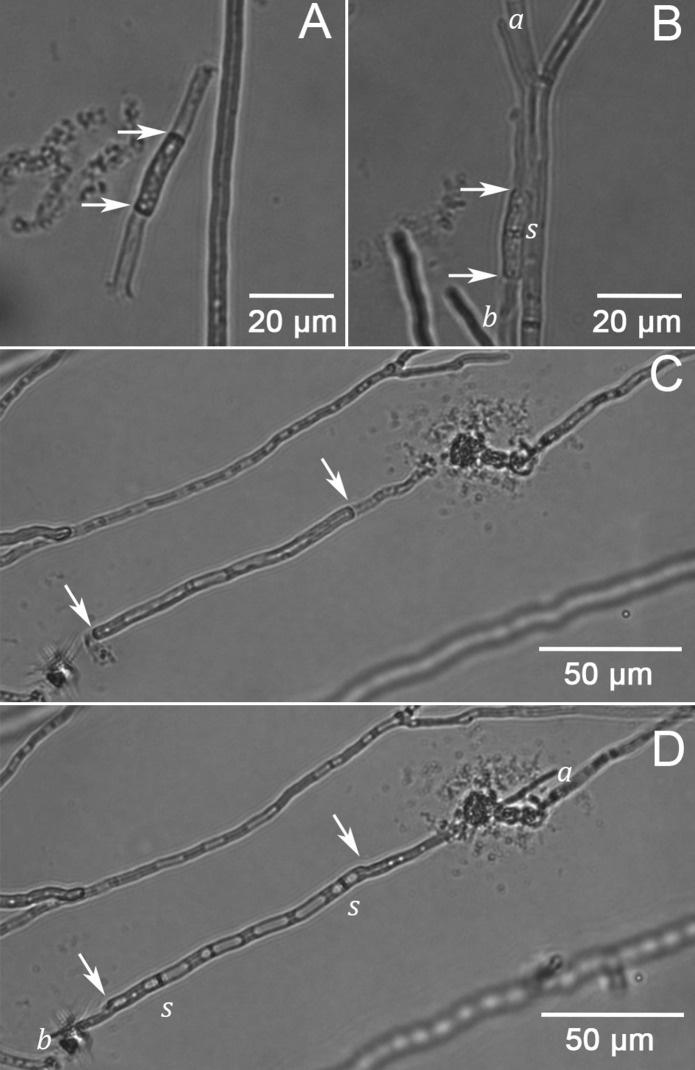
Single second compartments of *P. chrysogenum* (**A**, **B**) and *A. niger* (**C**, **D**) 60 (**A**, **C**) and 240 (**B**, **D**) min after laser dissection. Arrows denote the location of dissection, *s* the location of the septa and *a* and *b* the location of the apex of hyphae formed through the apical of basal septum, respectively

Growth from single (pseudo-)2nd compartments was recorded after dissection of hyphae both within the (pseudo-)apical and (pseudo-)3rd compartments. To this end, *R. stolonifer* was cut 500 and 1000 µm from the apex. After dissection, 42, 94, 57 and 0% of previously formed lateral branches *A. niger*, *P. chrysogenum, S. commune* and *R. stolonifer* (pseudo-)2nd compartments continued their growth. These branches had a mean extension rate of 14.1 ± 12.4, 27.3 ± 8.9, and 11.4 ± 23.8 µm h^−1^ for *A. niger*, *P. chrysogenum* and *S. commune*, respectively (Table 2). Newly formed branches of single (pseudo-)2nd compartments grew through the apical or basal septum or were formed laterally. Lateral branches were formed in 8, 2 and 50% of the cases in *A. niger, P. chrysogenum* and *S. commune*, respectively. Branches growing through the apical septum of the 2nd compartments were formed in 69, 60 and 45% of the 2nd compartments of *A. niger, P. chrysogenum* and *S. commune*, respectively, while this was 43, 37 and 36% for basal septa. In total, 79, 70 and 55% of single 2nd compartments resumed growth by forming a branch through either apical, basal, or both septa for *A. niger, P. chrysogenum* and *S. commune*, respectively (Fig. 1). Growth rates of newly formed branches of single 2nd (pseudo-)compartments was similar to the growth speed of branches that had only been dissected in the apical compartment (Table 2). In the case of *R. stolonifer*, only lateral branches were formed from single 2nd compartments. These branches were observed in 17.2% of the single pseudo-2nd compartments and had a mean extension rate of 15.5 ± 14.4 µm h^−1^ (Table 2). This growth speed was about threefold lower than that of newly formed branches of hyphae that had only been dissected at the apex. Together, growth rate of single second compartments is lower than that of the apical compartment (Tables 1, 2). Thus, the second compartment is not self-sustaining in reaching the maximal hyphal growth rate as found at the hyphal apex.

### Predictors for continued growth of dissected hyphae

**Fig. 2:**
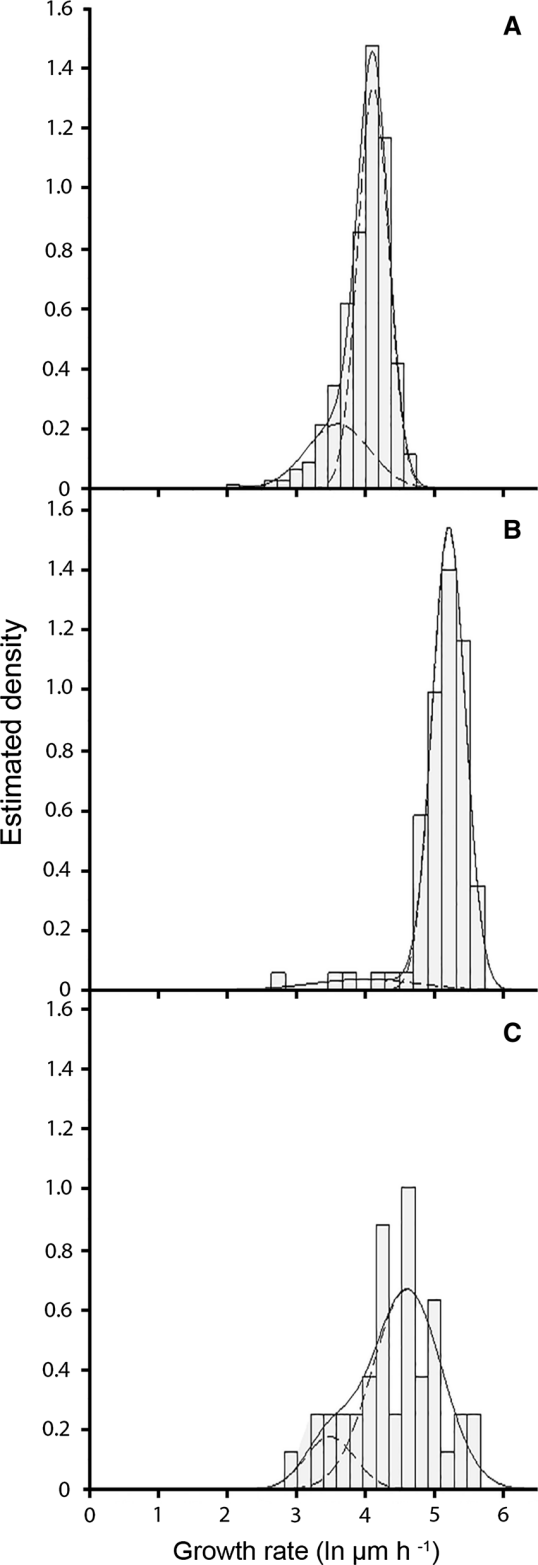
Distribution of growth rate of peripheral leading hyphae of *P. chrysogenum* (A), *S. commune* (B) and *R. stolonifer* (C)

Residual cytoplasmic volume of the apical compartment after dissection is a predictor of continued growth of *A. niger* after dissecting the second compartment (Tegelaar and Wösten 2017). Here, it was assessed whether growth rate, volume of the (pseudo-)apical compartment and/or the amount of cytoplasmic loss are predictors of continued growth of hyphal apices after dissection of *P. chrysogenum* and *R. stolonifer* in the second compartment. *S. commune* was not included since only 4% of its hyphae stopped growing after dissection of the 2nd compartment (see above) hampering statistical analysis of potential predictors of continued growth. Growth rate of *P. chrysogenum* and *R. stolonifer* hyphae was distributed unimodally (Fig. 2). Relative slow- or fast-growth within these unimodal distributions was not causally linked to continued growth after dissection of the second compartment of these fungi (Data not shown). The initial volume of *P. chrysogenum* apical compartments that continued or stopped growing was 2.6 ± 0.2 or 2.0 ± 0.3 picoliter (Table 3). This and the fact that absolute residual cytoplasmic volume in the apical compartment after dissecting the 2nd compartment was larger for continuing apical compartments (Table 3) shows that volume of the first compartment is a predictor of continued growth in the case of *P. chrysogenum*. Differences in absolute or relative cytoplasmic volumes of pseudo-apical compartments before or after laser dissection of the pseudo-2nd compartment of *R. stolonifer* were not found between hyphae that stopped or continued their growth. The same was found for single (pseudo-)2nd compartments of *P. chrysogenum* and *R. stolonifer* and also for *A. niger* (Table 3). Together, these results show that absolute cytoplasmic volume is a predictor of continued growth of apical compartments of *P. chrysogenum,* while in the case of *A. niger* the relative residual cytoplasmic volume after dissection is causally linked with continued growth (Tegelaar and Wösten 2017).

**Table 3:** Volumes of single (pseudo-)apical or single (pseudo-)2nd compartments before and after laser dissection of (pseudo-)2nd compartments and (pseudo-)apical and (pseudo-)3rd compartments, respectively. ^a^Data from Tegelaar and Wösten (2017)

	Volume before laser dissection (pl)	Volume after laser dissection (pl)
*Aspergillus niger*		
Stopping apical compartment^a^ N = 6	6.4 ± 1.7	3.9 ± 1.8
Continuing apical compartment^a^ N = 43	9.2 ± 1.3	8.1 ± 1.4
Stopping second compartment N = 12	0.9 ± 0.3	0.9 ± 0.3
Continuing second compartment N = 62	0.9 ± 0.1	0.9 ± 0.1
*Penicillium chrysogenum*		
Stopping apical compartment N = 31	2 ± 0.3	1.8 ± 0.4
Continuing apical compartment N = 53	2.6 ± 0.2	2.5 ± 0.2
Stopping second compartment N = 17	0.6 ± 0.1	0.6 ± 0.1
Continuing second compartment N = 40	0.6 ± 0.2	0.6 ± 0.2
*Rhizopus stolonifer*		
Stopping pseudo-apical compartment N = 11	6.1 ± 2.4	2 ± 2.8
Continuing pseudo-apical compartment N = 13	4.8 ± 1.1	3 ± 0.8
Stopping pseudo-second compartment N = 16	5.1 ± 1.1	2.6 ± 0.9
Continuing pseudo-second compartment N = 6	6.4 ± 3.8	2.2 ± 1.7

## Discussion

Fungal hyphae have been reported to require a minimal length or minimum number of compartments to maintain their growth rate (Trinci 1971). For instance, the peripheral growth zones of *A. niger* and *P. chrysogenum* would consist of 11 and 13 intact compartments and 8660 µm for the non-septate hyphae of *R. stolonifer.* However, it was shown that the apical compartment of *A. niger* can maintain its own growth rate (Tegelaar and Wösten 2017). Thus, sub-apical compartments are not needed to support growth of the apical compartments of *A. niger* hyphae but were rather shown to function as back-up to maintain peripheral growth in the case of apical injury. Here, it was shown that (pseudo-)sub-apical compartments are also not needed to support growth of (pseudo-)apical compartments of the basidiomycete *S. commune,* the ascomycete *P. chrysogenum* and the mucoromycete *R. stolonifer.* Like *A. niger*, these sub-apical compartments function as a back-up system in the case of apical injury. In addition, it is shown that in contrast to the apical compartment, the second compartment is not self-sustaining to reach the maximal growth rate of the (pseudo-)apical compartment.

A total of 9, 4, and 40% of the (pseudo-)apical compartments of *P. chrysogenum, S. commune* and *R. stolonifer* stopped growing after dissection, respectively. The *P. chrysogenum* hyphae that continued growing after dissection of the second compartment lost only 1% of their cytoplasm, while losses of 13 and 27% were found for *S. commune* and *R. stolonifer*, respectively. Moreover, in agreement with Tegelaar and Wösten (2017) a closed state of the apical septum in *P. chrysogenum* and *S. commune* was found not to be a significant contributor to the successful continuation of apical growth after dissection of the second compartment. In fact, *R. stolonifer* does not have septa in its vegetative hyphae but those hyphae that continued growth after dissection still maintained their initial growth rate.

The property of single (pseudo-)apical compartments to continue their growth raised the question if (pseudo-)2nd compartments also have this potential. This was assessed by cutting in both the first and third compartment or, in the case of *R. stolonifer* by cutting 500 and 1000 µm from the apex. Growth from single (pseudo-)2nd compartments took place from existing or newly formed branches. The branches were formed laterally, or grew through the apical or basal septum. Apical growth of the branches of the 2nd single (pseudo-)compartments was about fourfold (*R. stolonifer*), 13-fold (*P. chrysogenum*), 11-fold (*A. niger;* Tegelaar and Wösten 2017) and tenfold (*S. commune*) lower when compared to single (pseudo-)apical compartments. This lower growth rate may be caused by decreased turgor pressure (Lew 2011) or loss of Ca^2+^ potential (Lew 1999), secretory vesicles (Takeshita et al. 2017) or proteins (Lichius et al. 2012). In addition, the medium surrounding the detached (pseudo-)2nd compartments may be reduced in nutrients when compared to the medium the apical compartments are exposed to.

The fact that pseudo-apical and even single pseudo-second compartments of *R. stolonifer* can continue growing after being isolated from the rest of the mycelium is remarkable. Intuitively, one would assume that the pseudo-apical and pseudo-second compartments would lose their cytoplasm after dissection. However, total loss of cytoplasm occurs with low incidence, if at all. This may be explained by the capillary force of the hyphae and by organelles like vacuoles that block the opening(s) of the dissected compartments. Clearly, the presence of septa promotes the incidence of hyphal survival after dissection. However, the finding that growth of pseudo-apical septa is still observed after loss of 27% of the cytoplasm suggests that *R. stolonifer* has evolved mechanisms to maintain growth after loss of a relatively high percentage of cytoplasm. Such mechanisms seem to be absent in ascomycetes and basidiomycetes and would partly compensate for the absence of septa in the aseptate fungi.
